# A nomogram for individually predicting the overall survival in colonic adenocarcinoma patients presenting with perineural invasion: a population study based on SEER database

**DOI:** 10.3389/fonc.2023.1152931

**Published:** 2023-05-19

**Authors:** Junhong Chen, Hao Zhou, Hengwei Jin, Kai Liu

**Affiliations:** Department of Hepatobiliary and Pancreatic Surgery II, General Surgery Center, The First Hospital of Jilin University, Changchun, China

**Keywords:** perineural invasion, nomogram, predict, overall survival, colonic adenocarcinoma

## Abstract

**Background:**

Colonic adenocarcinoma, representing the predominant histological subtype of neoplasms in the colon, is commonly denoted as colon cancer. This study endeavors to develop and validate a nomogram model designed for predicting overall survival (OS) in patients with colon cancer, specifically those presenting with perineural invasion (PNI).

**Methods:**

The Surveillance, Epidemiology, and End Results (SEER) database supplied pertinent data spanning from 2010 to 2015, which facilitated the randomization of patients into distinct training and validation cohorts at a 7:3 ratio. Both univariate and multivariate analyses were employed to construct a prognostic nomogram based on the training cohort. Subsequently, the nomogram’s accuracy and efficacy were rigorously evaluated through the application of a concordance index (C-index), calibration plots, decision curve analysis (DCA), and receiver operating characteristic (ROC) curves.

**Results:**

In the training cohorts, multivariable analysis identified age, grade, T-stage, N-stage, M-stage, chemotherapy, tumor size, carcinoembryonic antigen (CEA), marital status, and insurance as independent risk factors for OS, all with *P*-values less than 0.05. Subsequently, a new nomogram was constructed. The C-index of this nomogram was 0.765 (95% CI: 0.755–0.775), outperforming the American Joint Committee on Cancer (AJCC) TNM staging system’s C-index of 0.686 (95% CI: 0.674–0.698). Calibration plots for 3- and 5-year OS demonstrated good consistency, while DCA for 3- and 5-year OS revealed excellent clinical utility in the training cohorts. Comparable outcomes were observed in the validation cohorts. Furthermore, we developed a risk stratification system, which facilitated better differentiation among three risk groups (low, intermediate, and high) in terms of OS for all patients.

**Conclusion:**

In this study, we have devised a robust nomogram and risk stratification system to accurately predict OS in colon cancer patients exhibiting PNI. This innovative tool offers valuable guidance for informed clinical decision-making, thereby enhancing patient care and management in oncology practice.

## 1 Introduction

Colonic adenocarcinoma, representing the predominant histological subtype of neoplasms in the colon, is commonly denoted as colon cancer. This disease ranks as the third most commonly diagnosed malignancy and the second leading cause of cancer-related mortality in the United States, while also representing a significant global public health issue (1). The phenomenon of “perineural invasion” (PNI) describes the infiltration of tumors into nerve structures and their subsequent dissemination along nerve sheaths (2). Recognizing its clinical implications, the American Joint Committee on Cancer (AJCC) TNM system and the National Comprehensive Cancer Network (NCCN) guidelines have identified PNI as a prognostic factor and a high-risk factor, respectively (3). Consequently, the prognosis of colon cancer patients exhibiting PNI is of considerable concern.

In contemporary clinical practice, the AJCC TNM system serves as the predominant methodology for risk assessment in colon cancer, as well as the primary staging system utilized to evaluate the survival prognosis of affected patients (4, 5). Notably, the AJCC TNM staging system omits several pertinent clinical factors that may impact the prognosis of colon cancer, including tumor grade and carcinoembryonic antigen (CEA) levels (6, 7). Consequently, to enhance the reliability of survival predictions for patients with colon cancer, it is essential for clinicians to consider these additional factors in conjunction with the AJCC TNM system.

Recently, nomogram-based clinical models have gained prominence in predicting various types of tumors (8–10). These models not only integrate intricate prognostic factors but also facilitate the calculation and prediction of survival rates for individual patients using a straightforward numerical valuation system (11, 12). Moreover, certain nomograms have demonstrated superiority over the AJCC TNM system in predicting tumor survival (13, 14). In the context of colon cancer, numerous researchers have developed pertinent clinical prediction models (15, 16). However, a nomogram for predicting overall survival (OS) in colon cancer patients with PNI has yet to be established. The present study aims to develop and validate a nomogram utilizing the Surveillance, Epidemiology, and End Results (SEER) database to predict OS in colon cancer patients with PNI, thereby enabling clinicians to provide accurate and personalized treatment recommendations.

## 2 Materials and methods

### 2.1 Cohorts formation and data collection

In the current study, the primary patient cohort was obtained from the SEER database, employing site codes C18.0-18.9. This cohort included individuals diagnosed with colonic adenocarcinoma, a specific type of colon cancer, between 2010 and 2015. Notably, all patients in the cohort presented with PNI at the time of diagnosis. The inclusion criteria comprised colon cancer patients identified by the International Classification of Diseases (ICD) codes: 8140, 8210, 8261, 8263, 8480, and 8490. Exclusion criteria consisted of the following (1): patients without a primary tumor; (2) insufficient information on histological grade, surgical procedures, tumor size, T-stage, N-stage, CEA levels, insurance, marital status, and PNI. After applying these screening criteria, 5,925 colon cancer patients with PNI were identified and subsequently divided into two cohorts: a training cohort comprising 4,149 patients (70%) and a validation cohort consisting of 1,776 patients (30%).

In this analysis, we incorporate a comprehensive set of variables, including: age, gender, race, grade, T-stage, N-stage, M-stage, surgery, radiotherapy, chemotherapy, tumor size, CEA levels, insurance, and marital status. The primary endpoint for our study is OS, defined as the time interval between the initial diagnosis and either the patient’s death or the last recorded follow-up.

### 2.2 Statistical analysis

In the present study, the selection of eligible patients was performed through randomization, employing the R software version 3.6.3. Subsequently, these patients were assigned to two distinct cohorts, in a 7:3 ratio, for the purpose of creating a training cohort, which encompassed 70% of the patients (n = 4149), and a validation cohort, comprising 30% of the patients (n = 1776). The training cohort was utilized for the construction of a nomogram model, which was based on a comprehensive analysis of the available data. Validation of the model was carried out by utilizing the independent validation cohort data, thus ensuring its generalizability and reliability.

The present study employed both univariate and multivariate analyses using IBM SPSS software (version 25.0) on a training cohort to identify risk factors influencing the OS prognosis. Based on the significant prognostic risk factors identified, a nomogram model for predicting the 3- and 5-year OS was developed using R software (version 3.6.3). The predictive performance of the nomogram model was evaluated using concordance index (C-index) and area under receiver operating characteristic curves (AUCs). Calibration plots were used to assess the calibration ability of the model in predicting the 3- and 5-year OS. Furthermore, decision curve analyses (DCAs) were conducted to evaluate the clinical practicability and net benefit of the nomogram. Additionally, a hazard stratification system was established to categorize patients into three risk groups (low, intermediate, and high risk) based on a predefined cut-off value.

The C-index, calibration plot, receiver operating characteristic (ROC) curve, and decision curve analysis (DCA) curve were computed using R software (v 3.6.3) and relevant packages. The optimal cut-off value for risk stratification was determined using X-Tile software (v 3.6.1). Furthermore, survival curves were generated using GraphPad Prism 8. The statistical analyses were conducted using IBM SPSS statistics, v 25.0 (SPSS, Inc.). The significance level was set at *P* < 0.05 for all statistical tests.

## 3 Results

### 3.1 Clinical characteristics of patients

**Figure 1:**
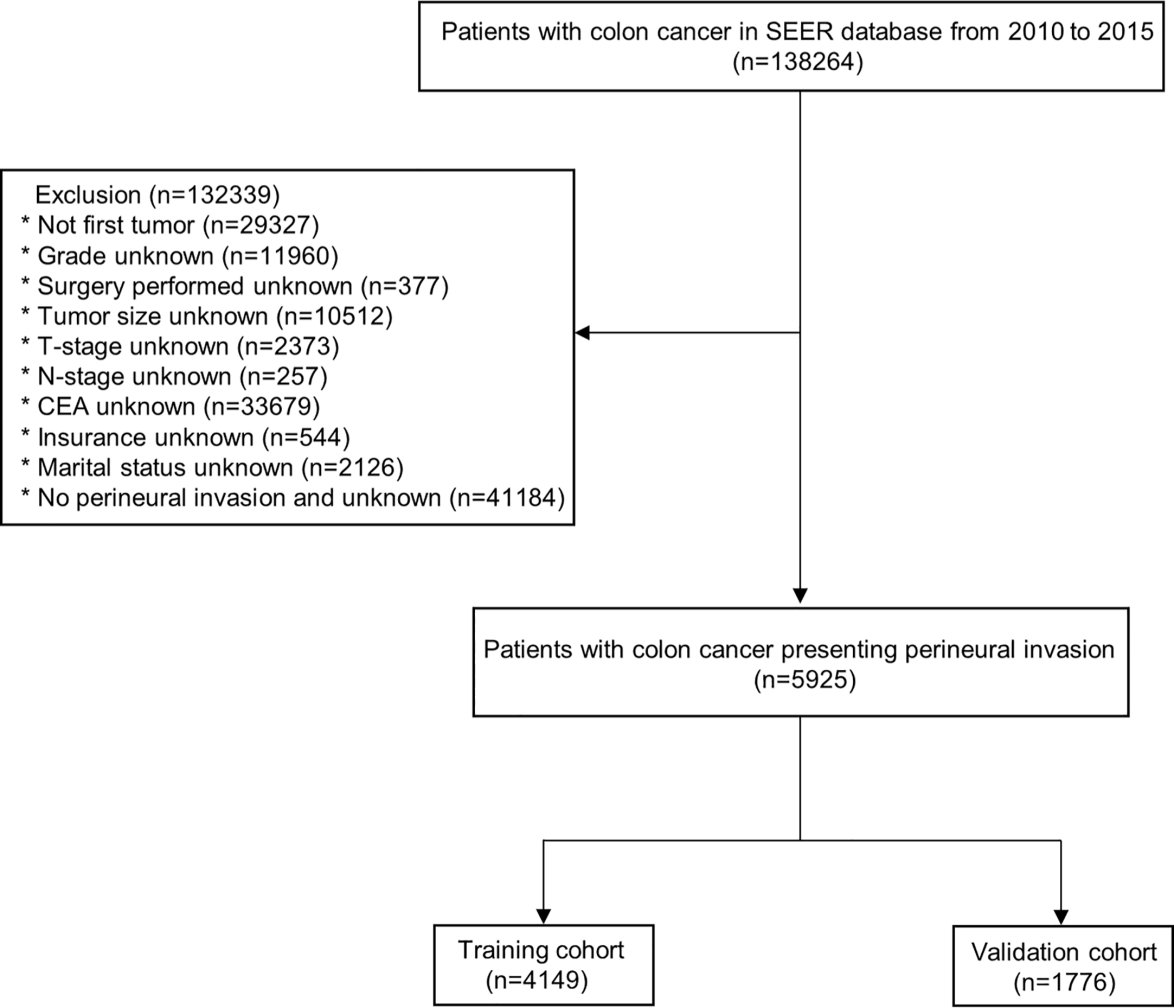
Flow diagram of screening colon cancer patients presenting with perineural invasion.

In this study, 5925 eligible colon cancer patients who presented with PNI were enrolled according to predefined screening criteria. These patients were categorized into two groups: a training cohort consisting of 4149 patients and a validation cohort consisting of 1776 patients (**Figure 1**). As indicated in **Table 1**, the enrolled colon cancer patients shared similar demographic and clinical characteristics. The age distribution was relatively balanced between patients aged less than 65 years and those aged 65 years or older, with a distribution of 51.5% and 48.5%, respectively. Additionally, the gender distribution was nearly equal, with 49.8% of patients being female and 50.2% being male. The majority of patients were white (75.6%), insured (80.5%), and married (54.7%). A significant proportion of patients had poor pathological grade II (61.9%) or III (28.4%), with grades I and IV only representing 3.2% and 6.5% of patients, respectively. Furthermore, the majority of patients were classified as stage T3 (53.9%) or T4 (42.5%), while T1 and T2 stages represented only 0.8% and 2.8% of patients, respectively. Most patients (78.9%) had regional lymphatic metastasis (N1 and N2), and 62.3% had no distant metastasis (M0). In terms of tumor size, the majority of patients (87.9%) had a malignancy greater than 3.0 cm and elevated pretreatment CEA levels (59.3%). Almost all patients (99.8%) underwent surgery, and most patients (63.3%) received chemotherapy, while only a small fraction (2.7%) received radiotherapy.

**Table 1:** Baseline demographic and clinical characteristics of training and validation cohorts.

	All cohorts N = 5925	Training cohort N = 4149	Validation cohort N = 1776
Age			
< 65	3054 (51.5%)	2163 (52.1%)	891 (50.2%)
≥ 65	2871 (48.5%)	1986 (47.9%)	885 (49.8%)
Gender			
Female	2949 (49.8%)	2054 (49.5%)	895 (50.4%)
Male	2976 (50.2%)	2095 (50.5%)	881 (49.6%)
Race			
White	4477 (75.6%)	3132 (75.5%)	1345 (75.7%)
Black	849 (14.3%)	584 (14.1%)	265 (14.9%)
Other ^a^	599 (10.1%)	433 (10.4%)	166 (9.3%)
Grade			
I	191 (3.2%)	130 (3.1%)	61 (3.4%)
II	3670 (61.9%)	2572 (62.0%)	1098 (61.8%)
III	1680 (28.4%)	1181 (28.5%)	499 (28.1%)
IV	384 (6.5%)	266 (6.4%)	118 (6.6%)
T stage			
T1	49 (0.8%)	29 (0.7%)	20 (1.1%)
T2	166 (2.8%)	110 (2.7%)	56 (3.2%)
T3	3192 (53.9%)	2249 (54.2%)	943 (53.1%)
T4	2518 (42.5%)	1761 (42.4%)	757(42.6%)
N stage			
N0	1250 (21.1%)	844 (20.3%)	406 (22.9%)
N1	2118 (35.7%)	1477 (35.6%)	641 (36.1%)
N2	2557 (43.2%)	1828 (44.1%)	729 (41.0%)
M stage			
M0	3690 (62.3%)	2598 (62.6%)	1092 (61.5%)
M1	2235 (37.7%)	1551 (37.4%)	684 (38.5%)
Surgery			
No	10 (0.2%)	5 (0.1%)	5 (0.3%)
Yes	5915 (99.8%)	4144 (99.9%)	1771 (99.7%)
Radiotherapy			
No/unknown	5767 (97.3%)	4037 (97.3%)	1730 (97.4%)
Yes	158 (2.7%)	112 (2.7%)	46 (2.6%)
Chemotherapy			
No/unknown	2176 (36.7%)	1475 (35.6%)	701 (39.5%)
Yes	3749 (63.3%)	2674 (64.4%)	1075 (60.5%)
Tumor size			
<3cm	718 (12.1%)	508 (12.2%)	210 (11.8%)
3-5cm	2713 (45.8%)	1918 (46.2%)	795 (44.8%)
>5cm	2494 (42.1%)	1723 (41.5%)	771 (43.4%)
CEA			
Negative/normal	2414 (40.7%)	1690 (40.7%)	724 (40.7%)
Positive/elevated	3511 (59.3%)	2459 (59.3%)	1052 (59.3%)
Insurance			
Any Medicaid	883 (14.9%)	618 (14.9%)	265 (14.9%)
Insured	4770 (80.5%)	3331 (80.3%)	1439 (81.0%)
Uninsured	272 (4.6%)	200 (4.8%)	72 (4.1%)
Marital status			
Married	3241 (54.7%)	2313 (55.7%)	928 (52.3%)
Unmarried	2684 (45.3%)	1836 (44.3%)	848 (47.7%)

T, Tumor; N, Node; M, Metastasis; CEA, carcinoembryonic antigen.

a other includes American Indian/AK Native, Asian/Pacific Islander and unknowns.

### 3.2 Independent risk factors for OS in training cohort

As per the results of the training cohort, univariate analysis demonstrated that several risk factors such as age, grade, TNM stage, chemotherapy, tumor size, CEA, marital status, and insurance had a significant association with OS (*P* < 0.05). Furthermore, the multivariate analysis identified that age, grade, TNM stage, chemotherapy, tumor size, CEA, and marital status (*P* < 0.001) were independently associated with OS. Additionally, insurance status was also identified as an independent risk factor (*P* = 0.029) for OS in the training cohort, as depicted in **Table 2**. These findings highlight the significance of various factors in predicting OS and suggest the need for targeted interventions for patients with identified risk factors.

**Table 2:** Univariate and multivariate analysis of colon cancer overall survival based on the training cohort.

	Univariate analysis		Multivariate analysis	
	Log rank χ2 test	*P*-value	HR (95%CI)	*P*-value
**Age**	85.866	< 0.001		< 0.001
< 65			reference	
≥ 65			1.448 (1.315-1.595)	
**Gender**	0.012	0.912	NI	
Female				
Male				
**Race**	3.420	0.181	NI	
White				
Black				
Other ^a^				
**Grade**	140.085	< 0.001		< 0.001
I			reference	
II			0.948 (0.724-1.241)	
III			1.365 (1.037-1.796)	
IV			1.506 (1.110-2.044)	
**T stage**	285.727	< 0.001		< 0.001
T1			reference	
T2			1.586 (0.612-4.109)	
T3			1.958 (0.806-4.755)	
T4			2.972 (1.222-7.227)	
**N stage**	258.274	< 0.001		< 0.001
N0			reference	
N1			1.677 (1.444-4.109)	
N2			1.733 (1.478-2.032)	
**M stage**	710.426	< 0.001		< 0.001
M0			reference	
M1			3.058 (2.763-3.384)	
**Surgery**	1.028	0.311	NI	
No				
Yes				
**Radiotherapy**	0.001	0.976	NI	
No/unknown				
Yes				
**Chemotherapy**	144.422	< 0.001		< 0.001
No/unknown			reference	
Yes			0.338 (0.306-0.374)	
**Tumor size**	97.624	<0.001		< 0.001
<3cm			reference	
3-5cm			1.121 (0.956-1.315)	
>5cm			1.329 (1.133-1.559)	
**CEA**	207.361	< 0.001		< 0.001
Negative/normal			reference	
Positive/elevated			1.401 (1.268-1.548)	
**Insurance**	9.125	0.010		0.029
Any Medicaid			reference	
Insured			0.880 (0.778-0.995)	
Uninsured			1.090 (0.869-1.366)	
**Marital status**	45.317	< 0.001		< 0.001
Married			reference	
Unmarried			1.242 (1.136-1.359)	

T, Tumor; N, Node; M, Metastasis; CEA, carcinoembryonic antigen; NI, not included in the multivariate survival analysis.

a other includes American Indian/AK Native, Asian/Pacific Islander and unknowns.

### 3.3 Nomogram construction and validation

**Figure 2:**
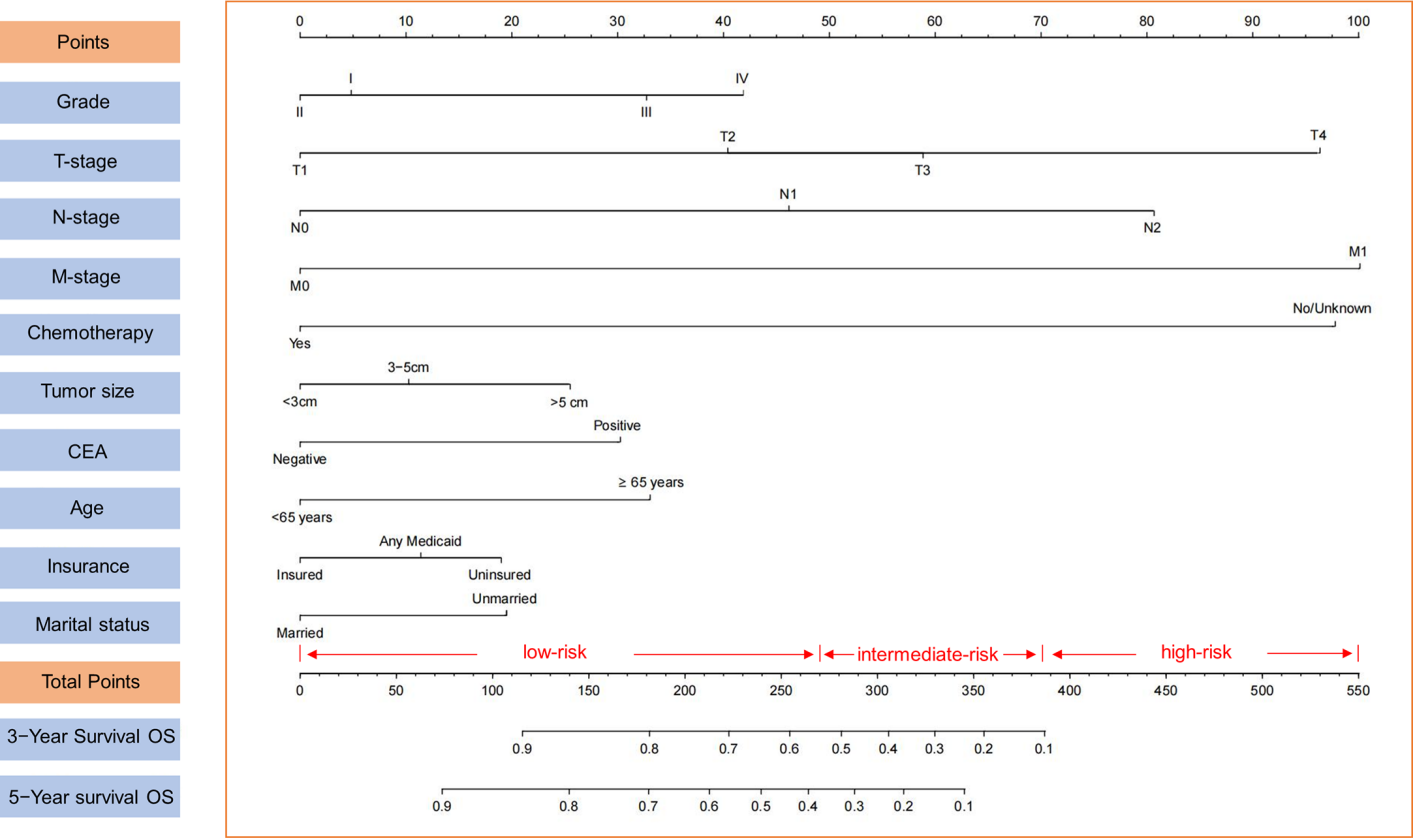
Nomogram for 3- and 5-year OS of colon cancer patients presenting with perineural invasion and risk stratification based on nomogram.

In this study, we developed a nomogram for predicting 3- and 5-year OS in colon cancer patients based on significant prognostic factors (**Figure 2**). The nomogram’s internal validity was assessed using C-index, calibration plots, ROC curves, and DCA curves. C-index values for the nomogram and AJCC TNM staging were compared in both the training and validation cohorts. The nomogram exhibited C-index values of 0.765 (95% CI: 0.755–0.775) and 0.773 (95% CI: 0.757–0.789) in the training and validation cohorts, respectively, whereas AJCC TNM staging yielded C-index values of 0.686 (95% CI: 0.674–0.698) and 0.694 (95% CI: 0.676–0.712) in the respective cohorts.

**Figure 3:**
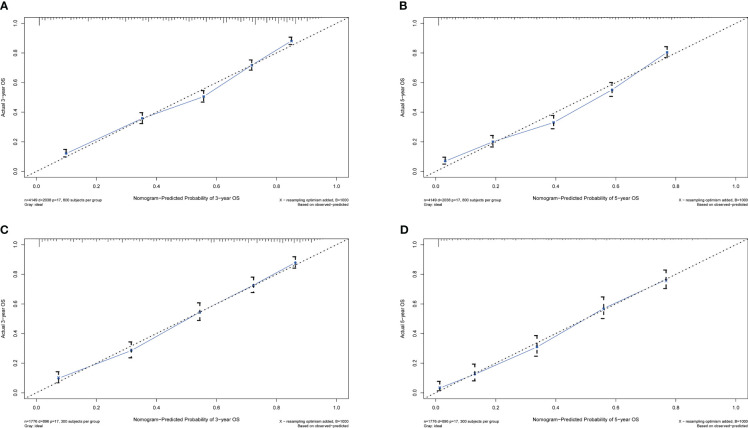
Calibration plots for 3- and 5-year OS in the training and validation cohorts. **(A)** Calibration plot of 3-year OS in the training cohort; **(B)** Calibration plot of 5-year OS in the training cohort; **(C)** Calibration plot of 3-year OS in the validation cohort; **(D)** Calibration plot of 5-year OS in the validation cohort.

**Figure 4:**
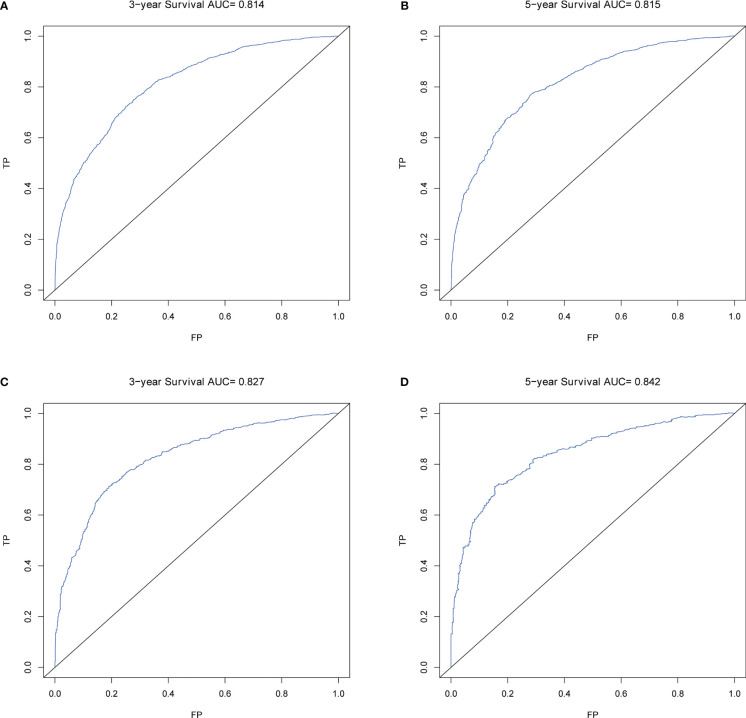
ROCs of OS for nomogram. **(A)** ROC of 3-year OS in the training cohort; **(B)** ROC of 5-year OS in the training cohort; **(C)** ROC of 3-year OS in the validation cohort; **(D)** ROC of 5-year OS in the validation cohort.

**Figure 5:**
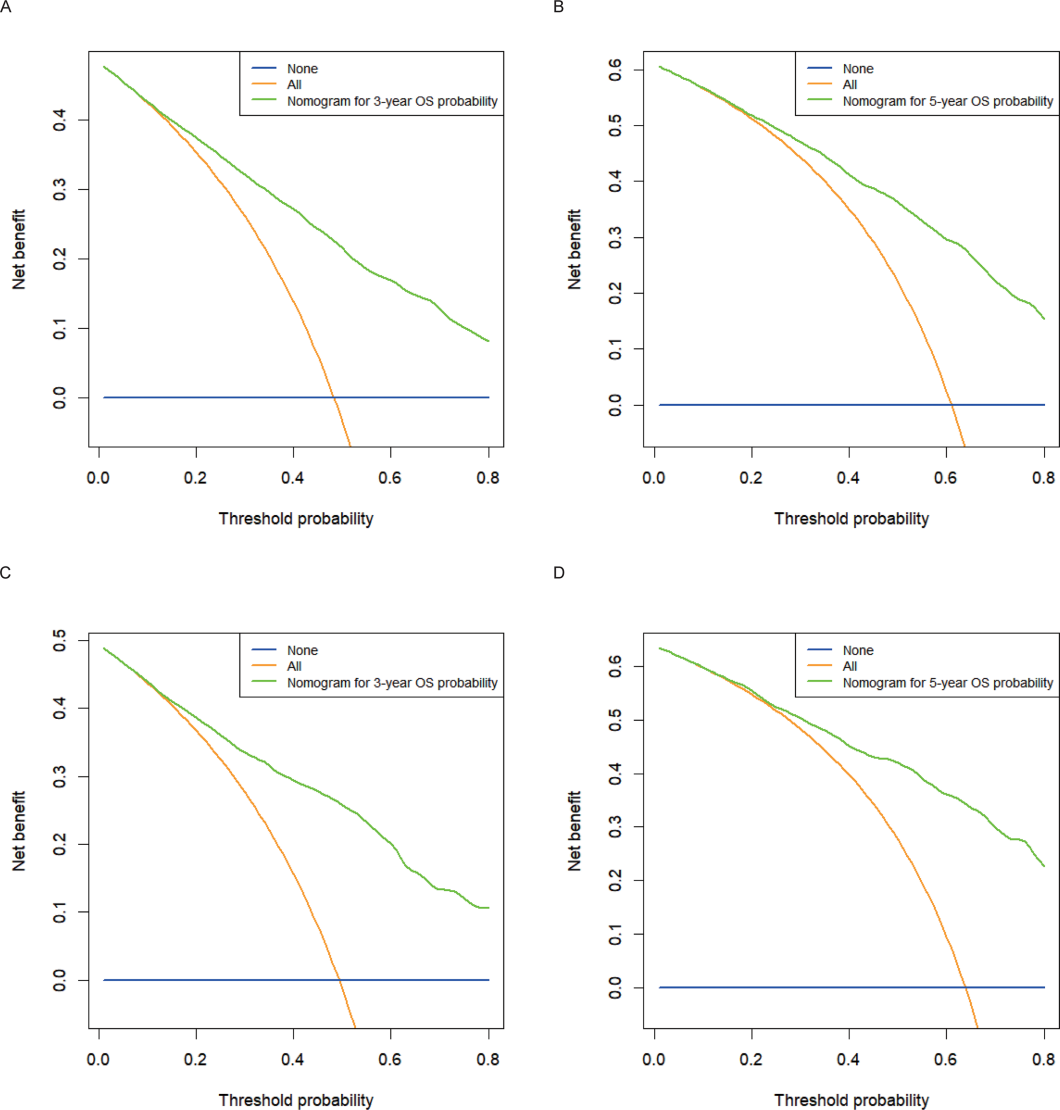
Decision curve analysis for 3- and 5-year OS in the training and validation cohorts. **(A)** DCA curve of 3-year OS in the training cohort; **(B)** DCA curve of 5-year OS in the training cohort; **(C)** DCA curve of 3-year OS in the validation cohort; **(D)** DCA curve of 5-year OS in the validation cohort.

Calibration plots for the probability of 3- and 5-year OS demonstrated that the nomogram’s predictions were more consistent with actual observations than TNM staging (**Figure 3**). Additionally, the 3- and 5-year ROC curves indicated a strong discriminative ability (**Figure 4**). To further evaluate the nomogram’s clinical utility, we constructed 3- and 5-year DCA curves for OS. The DCA curves demonstrated superior practicability and net benefit in both cohorts compared to TNM staging (**Figure 5**).

### 3.4 Risk stratification based on nomogram

**Figure 6:**
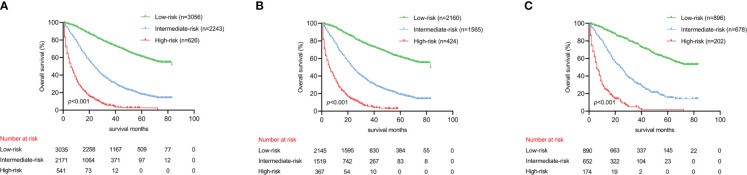
Kaplan-Meier analysis to different risk groups for OS in three cohorts. **(A)** OS for all cohorts; **(B)** OS for the training cohort; **(C)** OS for the validation cohort.

Utilizing optimal cut-off values for cumulative scores, a comprehensive risk stratification system was developed. This system effectively distinguished patients into three distinct categories: low-risk (score: 0–271), intermediate-risk (score: 272–386), and high-risk (score: 387–550) groups. As demonstrated in **Figure 6A**, the Kaplan–Meier analysis provided exceptional discrimination between these risk categories. Analogous findings were observed in both of the additional cohorts, as illustrated in **Figures 6B, C**.

## 4 Discussion

PNI is recognized as a significant high-risk factor and has been incorporated into the NCCN guidelines (3). A multitude of previous research has substantiated the influence of PNI on long-term outcomes in colon cancer (2, 17). Nevertheless, a limited number of studies have specifically concentrated on prognostic prediction for colon cancer patients presenting with PNI. Thus, it is of paramount importance to develop a precise clinical prediction model tailored for colon cancer patients with PNI, as this will enable medical professionals to make more informed and advantageous clinical decisions on behalf of their patients.

At present, the AJCC TNM system serves as the predominant method for estimating colon cancer patients’ survival prognosis. Despite regular updates to the TNM staging system occurring every 6 to 8 years, inaccuracies persist in the predictive accuracy of survival forecasts (18). Employing univariate and multivariate analyses, our study identified ten factors that significantly impacted OS, which included tumor grade, TNM stage, chemotherapy, tumor size, CEA levels, patient age, insurance status, and marital status. Furthermore, we developed and effectively validated a predictive nomogram model for 3- and 5-year OS, incorporating these significant factors to enhance the prognostic accuracy for colon cancer patients exhibiting PNI.

In the current investigation, a comprehensive examination of colon cancer patients presenting with PNI demonstrated that histological grade plays a significant role in determining prognosis. This finding aligns with the outcomes of earlier research (19). Intriguingly, our analysis revealed that patients exhibiting grade II pathology experienced more favorable prognoses compared to those with grade I, which diverges from prior studies. The disparity in inclusion criteria may account for this discrepancy. While previous research did not consider PNI status as a determinant for inclusion (16), our investigation specifically targeted colon cancer patients with PNI. This factor may elucidate the divergence in outcomes; however, a more precise rationale necessitates further inquiry and investigation. Tumor size, often considered an intuitive indicator of malignancy, also serves as a partial reflection of prognosis. In our study, tumor size was identified as a predictive variable, a finding corroborated by previous research (20).

In patients with colon cancer, Fergal et al. demonstrated that CEA serves as an independent prognostic factor (21). The study reported a 62% increased risk of mortality in patients with elevated CEA levels compared to those with normal levels (HR=1.62, 95% CI: 1.53–1.74) (21). Our investigation corroborates these findings, indicating that patients with elevated CEA levels (CEA-positive) experience poorer survival outcomes. Previous research has suggested that adjuvant chemotherapy may improve survival rates for colon cancer patients (22, 23). Notably, both the NCCN and the American Society of Clinical Oncology (ASCO) recommend postoperative adjuvant chemotherapy as a potentially beneficial treatment for such patients (24). Our study supports these recommendations.

Non-tumor related factors have been found to be associated with outcomes in colon cancer patients. One of these factors is age, with patients under the age of 65 having better results compared to their elderly counterparts. The increased prevalence of underlying cardiovascular and respiratory diseases in elderly patients may contribute to their poorer outcomes (25). Watanabe-Galloway et al. found that elderly patients (age ≥ 65 years) with colon cancer had significantly shorter survival (26). Insurance status is another important factor, with patients who have health insurance or Medicaid having better survival outcomes due to their increased access to healthcare. A previous study reported that uninsured patients had worse survival compared to those with insurance (27). Marital status has also been identified as an independent predictor of OS, with unmarried patients having a greater risk of death compared to married patients. This finding has been consistent across multiple studies (28, 29).

Constructing a clinical prediction model with high accuracy is of utmost importance for doctors and patients. In this study, the present nomogram was found to outperform the 7th edition of AJCC TNM in predicting colon cancer patient survival. Despite these promising results, several limitations should be taken into consideration. Firstly, the retrospective nature of this study, based on the SEER database, unavoidably introduced selective bias. Secondly, although the internal validation showed satisfactory results, external validation using a sufficient sample size was not conducted. Thirdly, the lack of detailed treatment information, such as adjunctive therapy, chemotherapy regimen, and radiation dose, in the SEER database may have led to biased results. In the current study, the primary patient cohort was obtained from the SEER database, employing site codes C18.0-18.9.such as tumor budding, microsatellite status, and genetic mutations (PLAC1, TP53, and KRAS), which were not registered in the SEER database, could potentially impact the accuracy of the nomogram in predicting colon cancer patient survival.

## 5 Conclusions

As per the current clinical practice, the ability to predict the survival of colon cancer patients with PNI requires refinement. In response, a nomogram has been devised to predict the 3- and 5-year OS for colon cancer patients who presented with PNI. The efficacy of the nomogram has been assessed through internal validation, indicating its favorable performance. With its demonstrated reliability, this nomogram presents promising prospects for its application in clinical settings, thereby facilitating personalized medical approaches for colon cancer patients.

## Data availability statement

The original contributions presented in the study are included in the article/supplementary material. Further inquiries can be directed to the corresponding author.

## Ethics statement

Ethical review and approval was not required for the study on human participants in accordance with the local legislation and institutional requirements. Written informed consent for participation was not required for this study in accordance with the national legislation and the institutional requirements.

## Author contributions

All authors are solely responsible for the content and writing of the manuscript. In this study, all authors contributed significantly to the design, data collection, interpretation, and manuscript preparation and revision.
